# Retinal image enhancement based on color dominance of image

**DOI:** 10.1038/s41598-023-34212-w

**Published:** 2023-05-03

**Authors:** Priyadharsini C, Jagadeesh Kannan R

**Affiliations:** School of Computer Science and Engineering, Vellore Institute of Technology, Chennai, Tamilnadu 600127 India

**Keywords:** Image processing, Machine learning

## Abstract

Real-time fundus images captured to detect multiple diseases are prone to different quality issues like illumination, noise, etc., resulting in less visibility of anomalies. So, enhancing the retinal fundus images is essential for a better prediction rate of eye diseases. In this paper, we propose Lab color space-based enhancement techniques for retinal image enhancement. Existing research works does not consider the relation between color spaces of the fundus image in selecting a specific channel to perform retinal image enhancement. Our unique contribution to this research work is utilizing the color dominance of an image in quantifying the distribution of information in the blue channel and performing enhancement in Lab space followed by a series of steps to optimize overall brightness and contrast. The test set of the Retinal Fundus Multi-disease Image Dataset is used to evaluate the performance of the proposed enhancement technique in identifying the presence or absence of retinal abnormality. The proposed technique achieved an accuracy of 89.53 percent.

## Introduction

According to the vision 2019 world report by World Health Organization, out of the 2.2 billion estimated visually impaired population worldwide, 1 billion cases could have been treated or prevented from vision impairment^1^. The initial non-invasive procedure in a routine eye clinical setting captures retinal fundus images and analyzes the anomalies. Examination of the eye is an early indicator of other diseases like hypertension, diabetes, cardiovascular-related diseases, etc.^2–4^. Thus, screening and examination of the eye, along with proper treatment, aids in preventing vision loss and protection from the risk of other diseases.

Typical quality issues in fundus images are due to noise, illumination, contrast, and sharpened regions within the image. Ophthalmologists need to view the features of retinal images to suggest appropriate treatment. Improper lighting may result in dark or bright images leading to less visibility of anomalies^5^. To overcome the illumination or contrast issues on captured retinal images and aid in better visibility of anomalies, image enhancement is an essential step in the image pre-processing stage^6^. Existing enhancement algorithms are based on the following approaches- histogram-based approach, transformation-based approach, and filter-based approach^7^. Out of various histogram-based approaches, Contrast Limited Adaptive Histogram Equalization (CLAHE) is found to be effective^8–10^.The enhancement techniques are mainly applied based on either of the three listed methods– 1. color image to grayscale conversion and enhancement of grayscale image, 2. splitting of channels in color space (ex., Splitting BGR (Blue, Green and Red) color space to blue, green, and red channels) and performing enhancement on individual channel followed by merging enhanced channel, 3. Performing enhancement techniques directly on color space^11,12^. A.W. Setiawan et al. chose a green channel from the RGB (Red, Green and Blue) color model and applied CLAHE^5^. Alwazzan et al. applied the Weiner filter followed by CLAHE on the green channel and merged with the red and blue channels of the RGB color model^13^. Jin et al. converted the input image from RGB color space to Lab (L and ab components represent lightness and chromaticity, respectively) color space and applied CLAHE on normalized individual components of the Lab (also called CIELAB color space defined by the International Commission on Illumination (CIE)) color model^14^. The Related works section discusses more research works in this domain. Most of the existing retinal enhancement methods are focused towards improving the contrast by choosing green channel or luminosity channel. The color information of retinal image will vary depending upon the retinal diseases a patient is suffering from along with other image quality issues. Choosing only the green channel can result in losing information available in optic disc and information about anomalies like drusen, cotton wool spots etc. So, it is important to select the color channel that displays more artifacts depending on the color dominance of retinal image for performing enhancement.

The unique contribution of this paper is utilizing the relation between color spaces to identify color dominance in a retinal image. RGB and Lab color space is chosen for selecting the channel to be enhanced for efficient enhancement of fundus images. The variance information in the blue channel is calculated to identify the color dominance of the selected retinal image and accordingly choose either a* or b* channel from the Lab color space to enhance its artifacts. Existing research works consider the L channel primarily from the Lab space to enhance the contrast in fundus images. The other two channels are generally less explored for image enhancement. But in this research, the information in a* and b* channels is utilized to enhance the retinal image dataset of multiple diseases. Instead of considering the overall average value of performance metrics, this paper analyses the performance metrics concerning various retinal disease categories individually to understand the suitability of proposed image enhancement on multiple fundus diseases with different anomalies.

The organization of the rest of this paper is as follows -Sections “Related works” and “Proposed enhancement method” present the overview of related works and proposed enhancement methodology; the experiments and discussion of results are under sections “Results” and “Discussion”; and finally, the conclusion of this work is under Section “Conclusion”.

## Related works

Image enhancement is an essential process in the design of computer-aided diagnosis based solutions. Retinal images are more susceptible to image quality issues. Over the years, researchers have been experimenting with different enhancement methods to enhance the visibility of artifacts in fundus images. Gupta et al.^15^ proposed an enhancement technique by applying adaptive gamma correction on the luminosity channel of Lab color space, where the weights are calculated using the cumulative distribution of the histogram of input image pixels. The enhanced image contrast in Lab space is further improved by applying a quantile-based histogram. The authors achieved a PSNR value of 27.67, an SSIM value of 0.66 for quantile equals 3, and a PSNR of 28.40 and SSIM of 0.69 for quantile equals 5 on the MESSIDOR dataset. Mohammed et al.^16^ applied CLAHE on the normalized luminosity channel of Lab space after segmentation of the retinal region. The enhanced luminosity channel is rescaled and merged with the other two chromatic color channels of Lab color space. The authors achieved a PSNR of 24.42, a local contrast index of 0.57, and an Entropy of 5.63. Zhou et al.^7^ proposed enhancement on fundus images based on luminosity and contrast. The authors applied Gamma correction on the Luminance gain matrix obtained by converting from RGB to HSV channel. The resultant image is converted to Lab color space with an intermediate step conversion to RGB color. CLAHE is applied on the L channel, and the output is converted to RGB color space. From the private dataset of 4000 images, 961 poor-quality images were extracted and tested. The average image quality assessment improved from 0.0404 to 0.4565 for low-quality images. Kumar et al.^20^ followed a similar approach but applied a weighted average histogram on the luminosity channel instead of CLAHE. The authors assessed the enhancement using the metrics - Edge based contrast measure (EBCM), contrast-enhanced image quality (CEIQ), naturalness image quality evaluator (NIQE), visual saliency-induced index (VSI), and modified measure of enhancement (MEME). Navdeep et al.^17^ addressed the non-uniform illumination problem by proposing two radiance-based histogram equalization (RIHE-RRVE and RIHE-RVE) for retinal vessel enhancement where one is a recursive algorithm, and the other is non-recursive. A tuneable parameter is estimated to split the histogram into sub-bands and to calculate the radiance value. If the radiance value is less than the threshold, the histogram equalization technique is applied. Performance evaluation is estimated on various databases - DRIVE, STARE, CHASE using the measures entropy, PSNR, Euclidean measure, and visual quality inspection. Qureshi et al.^18^ converted the RGB image to CIECAM02 color space, and the lightness component of this color space is converted to grayscale. Texture features of the fundus image are enhanced by applying a non-linear contrast enhancement technique on the resultant grayscale image. The performance is evaluated on all the images of MESSIDOR and DRIVE datasets resulting in mean values of 4.60 Entropy, 23.78 PSNR, and 8.78 contrast-to-noise ratios. Dissopa et al.^19^ enhanced local image contrast by applying CLAHE on Lab space. Followed by histogram rescaling and stretching to standardize the brightness range to Hubbard’s brightness range of fundus images for different histogram clip limits. The performance is measured in terms of Quaternion structural similarity, Global contrast factor, and lightness order error values. In a technique by Wang et al.^21^ , the fundus image is decomposed into three layers: base, detail, and noise. Then, a visual adaption model is framed to perform non-illumination correction using a luminance map at the base layer, weighted fusion for enhancing the detail layer, and denoising at the noise layer. The authors calculated the Local contrast index and entropy measures for quantitative assessment. To improve the blurred retinal images, Xiong et al.^22^ applied techniques specific to background and foreground on 319 images. Background pixels are estimated using an illumination map and transmission map. Foreground pixels are enhanced and captured by applying the combination of Mahalanobis distance and entropy-based enhancement methods.

**Table 1:** Existing Image Enhancement Techniques.

Studies	Dataset	Method	Performance metrics
Gupta et al.^15^	MESSIDOR	Adaptive Gamma correction on Luminosity gain matrix and quantile-based histogram equalization on Lab color space.	For quantile equals 3, PSNR = 27.67 and SSIM = 0.66.
Mohammed et al.^16^	DRIVE	CLAHE on the Luminosity channel of the Lab color space.	PSNR = 24.42, Entropy = 5.64,local contrast index = 0.57
Navdeep et al.^17^	DRIVE, CHASE, STARE	Radiance indicator-based histogram equalization technique.	Entropy, PSNR, Euclidean Measure, Visual quality inspection
Zhou et al.^7^	MESSIDOR, Private Dataset	Gamma correction is applied on the Luminosity gain matrix of the HSVcolor space and CLAHE on the L channel of the Lab color space.	Quality assessment metric in the range of (0-1).
Qureshi et al.^18^	MESSIDOR, DRIVE	Non-linear contrast enhancement on J component of CIECAM02 color space.	PSNR, contrast-to-noise ratio, intensity variation, Entropy
Dissopa et al.^19^	DiaretDB0, STARE	Local image contrast enhancement followed by standardization to Hubbard’s brightness range.	Quaternion structural similarity, Global contrast factor, and lightness order error
Kumar et al.^20^	STARE	Brightness adjustment on value channel of HSV color space followed by weighted average histogram equalization.	CEIQ, VSI, NIQE, MEME, EBCM
Wang et al.^21^	DIARETDB0, DIARETDB1	Decomposition of fundus images into the base layer, details layer, and noise layer. At each layer, different enhancement techniques are applied based on the visual adaption model.	Local contrast index, Entropy
Xiong et al.^22^	DIARETDB0, DIARETDB1, Private dataset	Illumination correction and transmission map estimation to extract background and foreground to retinal images followed by enhancement on foreground pixels.	Local contrast index

Table 1 presents the overall comparative analysis of the discussed image enhancement techniques. The analysis shows that the current research works perform enhancement techniques mainly on the Luminosity channels. To the best of our knowledge, the current research works have not considered quantifying color information for performing image enhancement. In the proposed method, we quantified the spread of information present in color channels by calculating the variance and performed image enhancement techniques based on color information.

## Proposed enhancement method

**Figure 1:**
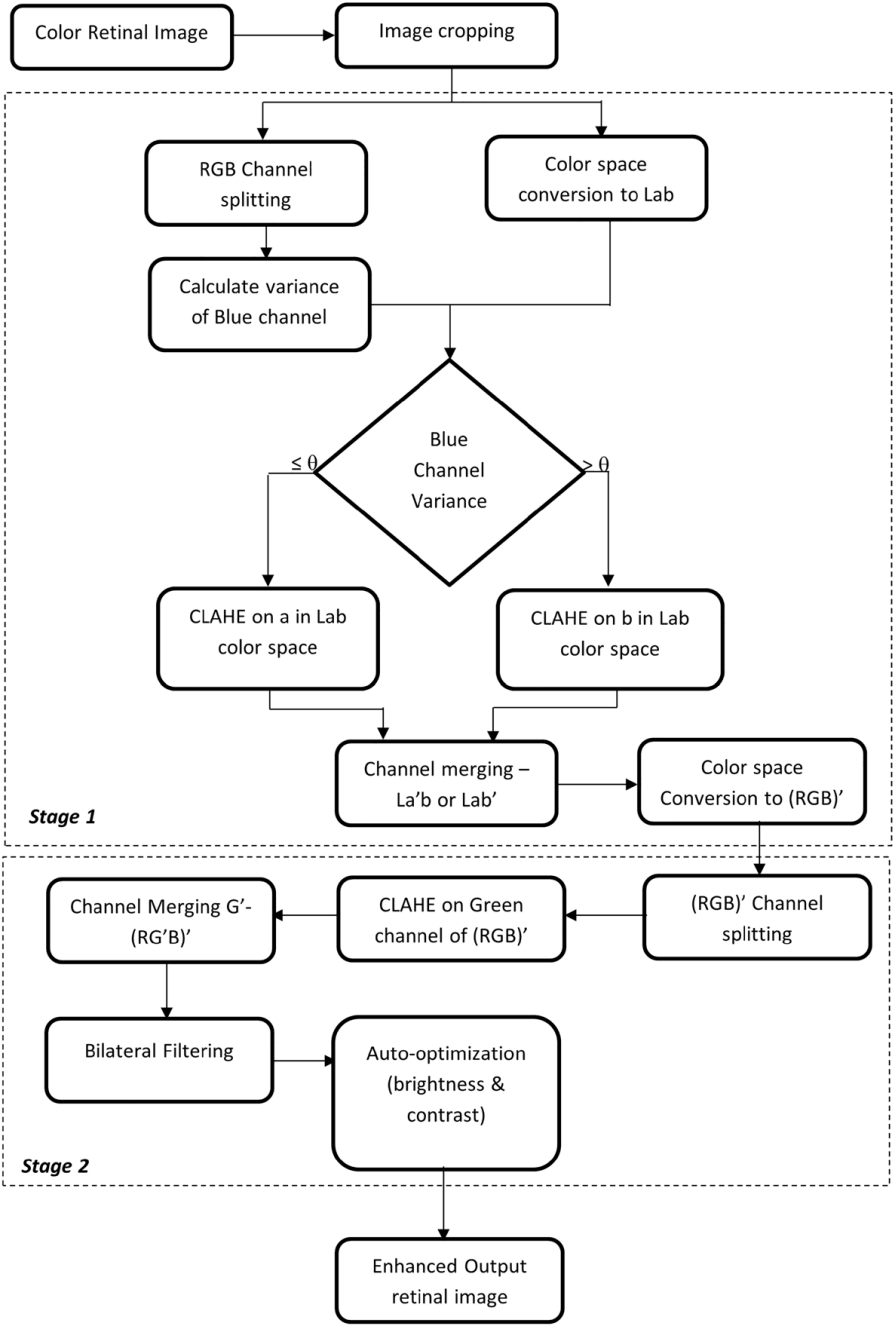
Flowchart of Proposed Method.

The proposed retinal fundus image enhancement method consists of two stages. Figure 1 presents the flow of each stage- Stage 1 and Stage 2. Stage 1 focuses on selecting the color channel for image enhancement, and Stage 2 focuses on noise removal, brightness, and contrast optimization.

### Fundus image dataset

**Figure 2:**
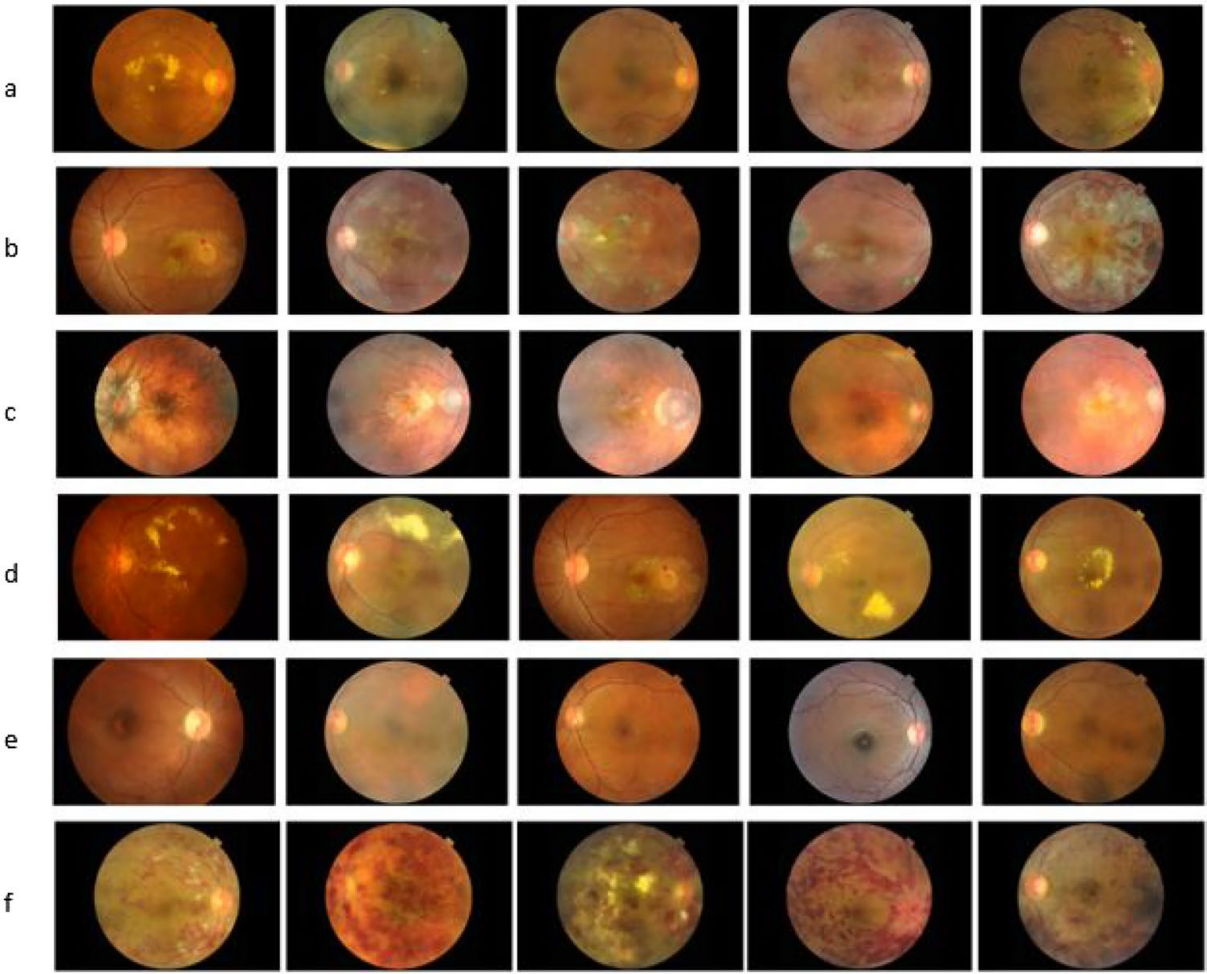
Sample images from the RFMiD dataset belonging to some of the categories. (**a**) Diabetic Retinopathy, (**b**) Chorioretinitis, (**c**) Myopia, (**d**) Exudation, (**e**) Retinal Pigment Epithelium Cells, (**f**) Central Retinal Vein Occlusion.

The Retinal Fundus Multi-Disease Image Dataset (RFMiD) dataset is chosen in this research to evaluate the performance of our proposed method because the database contains images of different color dominance, and the proposed method is also based on color dominance. RFMiD is more recent(published in 2021) and the only publicly available dataset that includes 45 retinal disease categories plus one set of healthy fundus images^23^. Figure 2. shows samples of retinal images from the RFMiD dataset with different resolutions and varied color dominance. The images are captured in either of three cameras with resolutions- 4288x2848, 2048x1536, and 2144x1424. The RFMiD dataset contains 1920 training images, 640 test images, and 640 validation images. This paper evaluates the effectiveness of the enhancement technique in binary retinal disease class prediction of fundus images into the normal or disease-affected fundus. Table 2 shows the distribution of healthy and unhealthy retinal images. To understand the suitability of the proposed enhancement method on other publicly available datasets, we tested the algorithm on DRIVE and MESSIDOR datasets and tabulated the results in Tables 7, 8 and 9.

**Table 2:** RFMiD dataset distribution.

Category	Training set	Validation set	Test set
Healthy fundus	401	134	134
Fundus images with retinal diseases	1519	506	506

### Stage 1

The input retinal image from the RFMiD dataset is of different resolutions. Image cropping is carried out for each retinal image by applying a square mask in the retinal region. The cropped retinal image in RGB color space is split into individual channels- red, green, and blue to calculate the variance of the blue channel using Eq. (1).

1 $$\begin{aligned} \sigma ^2 = {\frac{1}{mn}}{{\sum \limits _{i=0}^{m-1}}{\sum \limits _{j=0}^{n-1}}}{\left[ X_(i,j)-\mu \right] }^{2} \end{aligned}$$

where $X_(i,j)$, $\mu$, m and n represents individual pixels, mean, number of rows and columns in blue channel respectively.

The variance measure is selected to understand the spread of data variability. Table 3 shows the variance calculated for each channel from RGB, HSV, and Lab color space. Compared to the variance of all the channels listed in Table 3, the variance of the blue channel shows direct relation with the color dominance in fundus images. It is evident from the table that red dominant images have less blue variance while non-red dominant images have more blue variance value. The underlying principle of human vision and Lab color space is based on the opponent color model. Since Lab color space is based on human visual perception, Lab color space is chosen in this research. The a* channel in the Lab space denotes the relation between red and green pixel values in an image, while the b* channel depicts the yellow-blue pixel values in the Lab space. So, the retinal image in RGB color space is converted to Lab space using the transformations described in Eqs. (2)–(5)^24^. The conversion from RGB to Lab color space involves transformation to intermediate components X, Y and Z components- of CIE XYZ color space, and ${X_n}$, ${Y_n}$, ${Z_n}$ represents CIE XYZ tristimulus values of the reference white point.

2 $$\begin{aligned}{} & {} \begin{bmatrix} X\\ Y\\ Z \end{bmatrix} = \begin{bmatrix} 0.412453 &{} 0.357580 &{} 0.180423 \\ 0.212671 &{} 0.715160 &{} 0.072169 \\ 0.019334 &{} 0.119193 &{} 0.950227 \\ \end{bmatrix}. \begin{bmatrix} R \\ G \\ B \\ \end{bmatrix} \end{aligned}$$

3 $$\begin{aligned}{} & {} L = 116 f \left( \frac{Y}{Y_n} \right) - 16 \end{aligned}$$

4 $$\begin{aligned}{} & {} a^* = 500\left[ f \left( \frac{X}{X_n}\right) -f \left( \frac{Y}{Y_n}\right) \right] \end{aligned}$$

5 $$\begin{aligned}{} & {} b^* = 200\left[ f \left( \frac{Y}{Y_n}\right) -f \left( \frac{Z}{Z_n}\right) \right] \end{aligned}$$

where,

$$\begin{aligned} f(t) = \left\{ \begin{array}{ll} t^{(1/3)} &{} \text{ if } t >\left( \frac{6}{29}\right) ^3 \\ \\ \left( \frac{4}{29} + t \frac{1}{3} \left( \frac{29}{6}\right) ^2\right) &{} \text{ otherwise } \end{array} \right. \end{aligned}$$

**Table 3:** Variance Calculation in different color space.

Image	ID & Class	Variance of RGB color space			Variance of HSV color space			Variance of Lab color space		
		Blue	Green	Red	Hue	Saturation	Value	Luminosity	Red-Green value	Blue-Yellow value
	583 ODP	00091.77	01749.84	07257.04	00602.38	12433.39	07256.81	03376.63	00262.50	00618.58
	949 DR	00121.17	01379.79	04688.93	00455.48	11572.55	04688.70	02401.21	00152.82	00421.15
	918 MYA	00598.96	03905.07	09890.85	00952.09	09067.23	09890.61	05643.60	00172.70	00672.60
	133 EDN	00692.87	04614.02	08597.80	00410.97	08367.26	08597.46	05867.70	00040.79	00650.10
	665 ODE	00977.61	02945.45	05955.26	00567.07	06290.02	05955.09	04011.76	00061.48	00297.64
	259 RS	01868.32	03935.16	06437.54	00545.01	04165.14	06437.40	04869.92	00038.44	00202.35
	345 CRS	02199.51	02871.88	03615.08	01346.86	01089.59	03623.33	03419.37	00011.24	00040.80
	401 RS	03213.49	05440.53	07728.81	00192.86	02691.01	07728.98	06378.86	00030.50	00148.27
	213 MS	04262.72	08271.31	12670.32	00566.99	04403.10	12674.52	09530.19	00055.26	00369.09
	464 ERM	04404.08	06200.27	10199.85	00143.86	02164.67	10199.38	07622.49	00060.11	00109.42

After repeated experiments on analyzing the relation between blue variance and image color dominance, a threshold of $\theta =1500$ is fixed. For the red dominant image, i.e., with blue variance $\sigma ^2 \le \theta$, CLAHE is applied on a* channel of Lab space. And for the non-red dominant images with $\sigma ^2 > \theta$, CLAHE is applied on the b* channel of Lab space. The enhanced channel of Lab space is merged with the other two unchanged channels of Lab space and finally converted to RGB. The transformation from Lab to RGB color space involves intermediate conversion to CIE XYZ color space described in the Eqs. (6)–(9)^24^:

6 $$\begin{aligned}{} & {} X= X_n f^{-1} \left( \frac{L +16}{116} + \frac{a}{500} \right) \end{aligned}$$

7 $$\begin{aligned}{} & {} Y= Y_n f^{-1} \left( \frac{L +16}{116} \right) \end{aligned}$$

8 $$\begin{aligned}{} & {} Z= Z_n f^{-1} \left( \frac{L +16}{116} - \frac{b}{200} \right) \end{aligned}$$

where,

9 $$\begin{aligned}{} & {} f^{-1}(t) = \left\{ \begin{array}{ll} t^{3} &{} \text{ if } t > \frac{6}{29} \\ \\ 3(\frac{6}{29})^{2}\left( t-\frac{4}{29}\right) &{} \text{ otherwise } \end{array} \right. \nonumber \\{} & {} \begin{bmatrix} R\\ G\\ B \end{bmatrix} = \begin{bmatrix} 3.240479 &{} -1.537150 &{} -0.498535 \\ -0.969256 &{} 1.875992 &{} 0.041556 \\ 0.055648 &{} -0.204043 &{} 1.057311 \\ \end{bmatrix}. \begin{bmatrix} X \\ Y \\ Z \\ \end{bmatrix} \end{aligned}$$

where R, G, and B are components in RGB color space; X, Y, and Z are components in CIE XYZ color space, and ${X_n}$, ${Y_n}$, ${Z_n}$ represents CIE XYZ tristimulus values of the reference white point.

The output obtained by implementing algorithm 1 on the input fundus image is passed to the next phase : Stage 2.

### Stage 2

Stage 2 is focused on noise removal and brightness and contrast optimization on the output image from stage 1. Out of the red, green, and blue channels in the RGB color space, the green channel is chosen for further performance improvement because the green channel is proportional to the L channel of the Lab color space^7^. The Green channel has better visibility of more artifacts than the other two channels and is less noise-prone. So, in this research, the modified (RGB)’ channel from stage 1 is split, and CLAHE is applied on the green channel. The enhanced green channel is merged with the red and blue channels. Due to repeated enhancements, it is essential to perform noise removal. A bilateral filter is applied to perform noise removal using the Eq. (10) where the output image ($X_{output}$) is given by the weighted average of pixels in input image ($X_{input}$)^25^.

10 $$\begin{aligned} X_{output}[j]=\sum _i \frac{w_{ij}}{\sum _i w_{ij}} X_{input}[i] \end{aligned}$$

where,

$$\begin{aligned} w_{ij}= exp \left( -\frac{\vert \vert p_i - p_j \vert \vert ^2}{2\sigma ^2_{d}} \right) .exp \left( -\frac{(X_{input}[i]-X_{input}[j])^2}{2\sigma ^2_{r}} \right) \end{aligned}$$

where weight $w_{ij}$ is the product of photometric and euclidean distance between pixels $X_{input}[i]$ and $X_{input}[j]$ and $p_i$ denotes the position of $i{\textrm{th}}$ pixel

A bilateral filter is better than a popular Gaussian filter because of the algorithm characteristics to preserve edges between the regions in an image and reduce noise by applying a non-linear function over the image pixels. The brightness and contrast of a filtered image are auto-optimized by calculating the gain parameter ($\alpha$) and the bias parameter ($\beta$) using Eqs. (11)–(13). Alpha and beta values are auto-calculated specific to each input image to produce the final enhanced retinal fundus image.
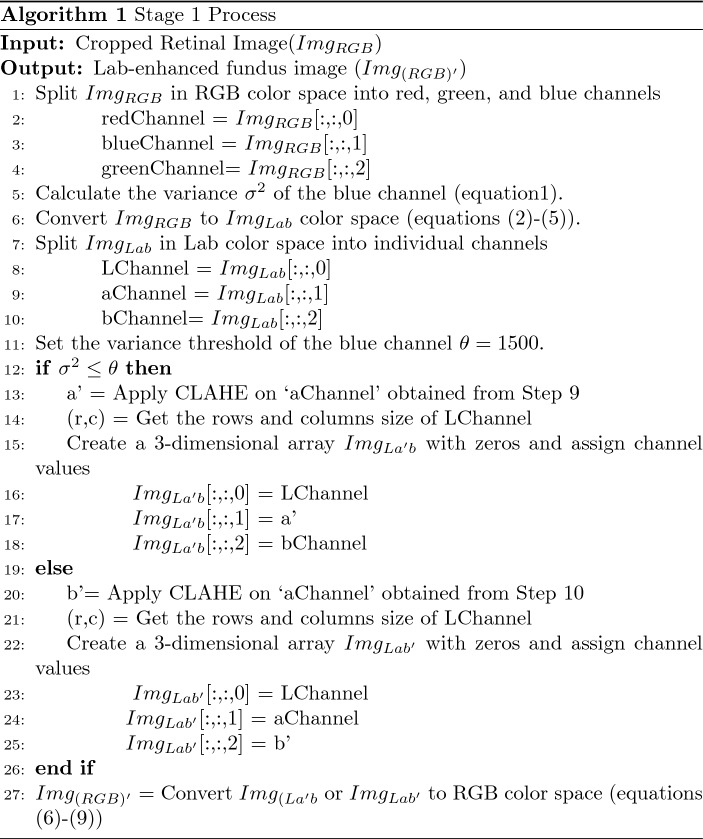

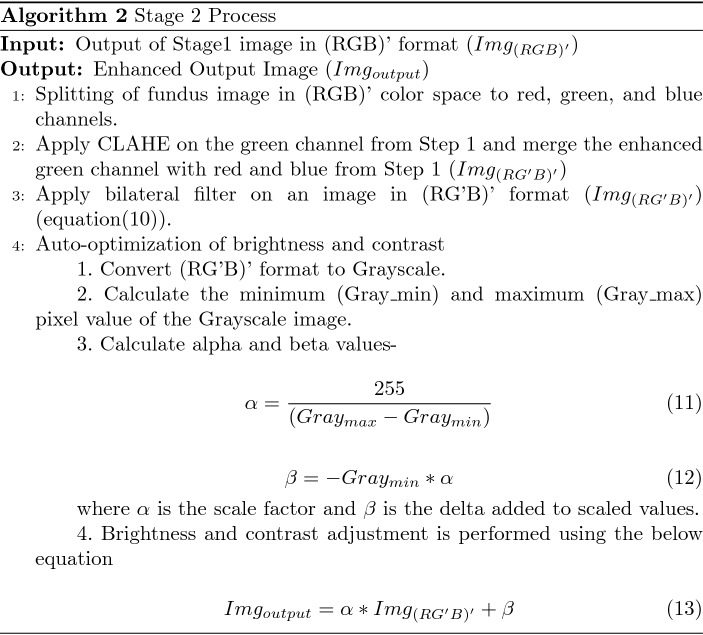


### Method evaluation

The proposed enhancement technique is applied to the RFMiD image data-set to test the disease prediction accuracy. Pre-trained VGG16 model using the transfer learning technique is trained with a training data-set (1920 images), validated with a validation data-set (640 images), evaluated on a test set (640 images), and the accuracy is estimated. A fully connected layer with 512 nodes and a Relu activation function, followed by a dropout layer, and a final layer with 1 node and a sigmoid activation function are added on top of the VGG16 model. The model is trained with a Stochastic gradient optimizer, and for regularization, dropout is used. The code for the proposed method was developed in python, and the VGG16 model was trained on Power Ai 9 server with 16 GB RAM at 8Hz. The experiment results are analyzed and discussed in the following section.

## Results

**Figure 3:**
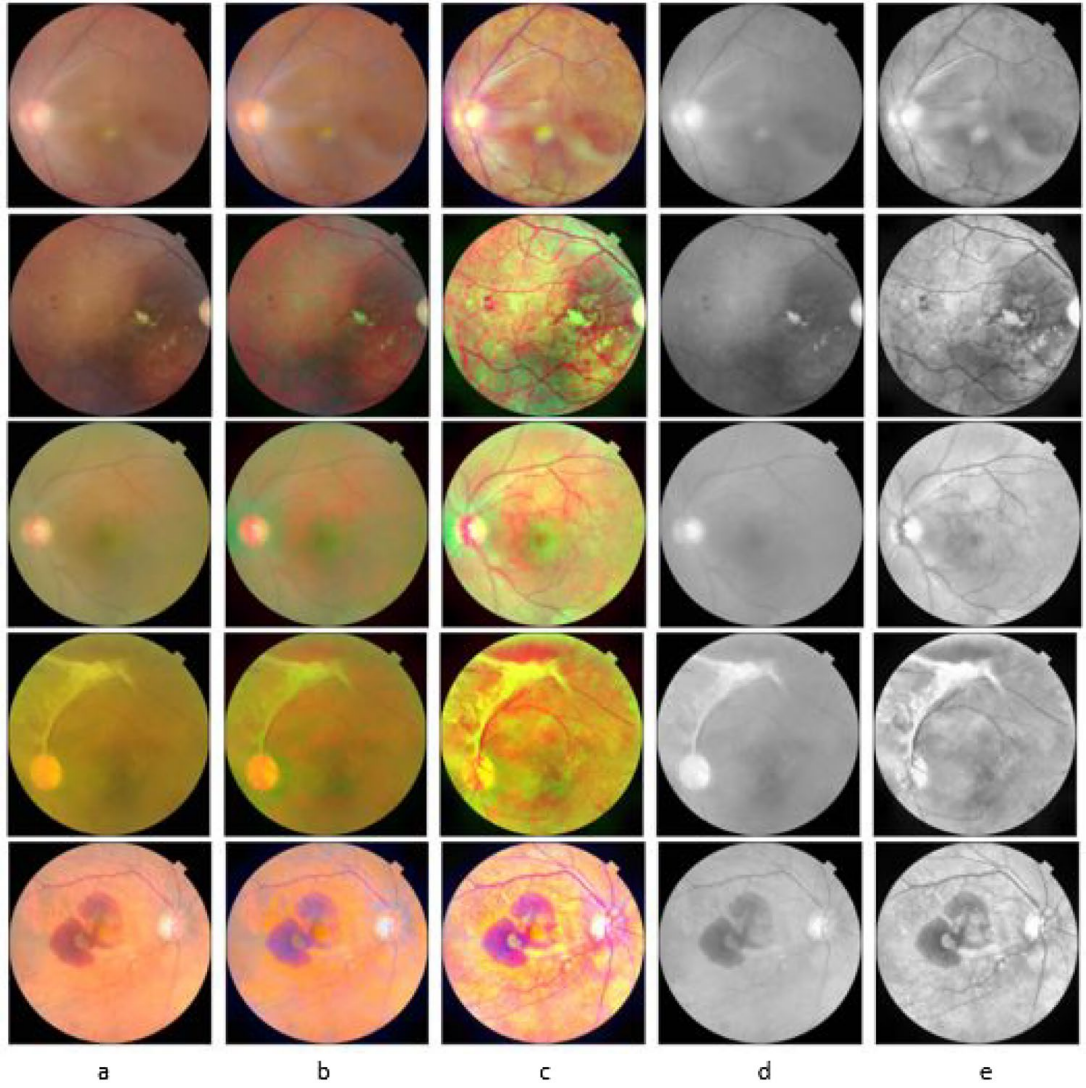
(**a**) Input image in RGB color space, (**b**) Output of Stage 1, (**c**) Output of stage 2, (**d**) Input image in grayscale, (**e**) Final output in Grayscale.

The proposed enhancement technique is implemented, trained, and validated on the training and validation set of the RFMiD data-set using a pre-trained VGG16 model and evaluated on the test set to identify the presence or absence of retinal abnormalities. The model performance is evaluated by calculating accuracy and F1 score. Accuracy is calculated as the ratio between the total number of correct predictions to the total number of predictions. F1 score is defined as the harmonic mean of precision and recall. Visual image analysis of the result is carried out in RGB color space and as well as in gray-scale. Figure 3 shows the comparison of the original input, stage 1 output, and stage 2 output in color space, along with a comparison between the gray-scale of the input image vs. the output of the proposed technique.

**Table 4:** RFMiD training dataset distribution.

Disease	Sample Count	Disease	Sample Count
Healthy Fundus (HF)	401	Diabetic Retinopathy (DR)	376
Media Haze (MH)	317	Optic disc cup (ODC)	282
Tessellation (TSLN)	186	Drusen (DN)	138
Myopia (MYA)	101	Optic Disc Pallor (ODP)	65
Optic disc Edema (ODE)	58	Laser Scars (LS)	47
Chorioretinitis (CRS)	32	Macular Scar (MS)	15
Exudation (EDN)	15	Epiretinal Membrane (ERM)	14
Retinal Traction (RT)	14	Parafoveal Telangiectasia (PT)	11
Macular Hole (MHL)	11	Tortuous Vessels (TV)	6
Retinitis Pigmentosa (RP)	6	Optociliary shunt (ST)	5
Asteroid Hyalosis (AH)	16	Retinitis (RS)	43
Age-Related Macular Degeneration (ARMD)	100	Branch Retinal Vein Occlusion (BRVO)	73
Central Serous Retinopathy (CSR)	37	Central Retinal Vein Occlusion (CRVO)	28
Retinal Pigment Epithelium Cells (RPEC)	22	Anterior Ischemic Optic Neuropathy (AION)	17
OTHER	34		

Table 4 tabulates the distribution of retinal disorders in the RFMiD training data-set. The performance of the enhancement technique is evaluated on the training set of the RFMiD data-set in terms of the following metrics - Mean square error (MSE), Peak-to-signal noise ratio (PSNR), and Universal Quality Index (UQI). UQI calculates correlation loss, distortion in contrast, and luminance as a single performance metric^26^. The similarity of the original input image and the enhanced image is evaluated using the Structural similarity index measure (SSIM), and Pearson correlation coefficient^27^, and the information variability of input and the enhanced image is estimated by comparing the Shannon entropy of the original and enhanced image. The higher Shannon entropy value indicates high information variability in an image. The considered metrics are calculated using the Eqs. (14)–(19) for each channel of RGB color space and averaged; the results are tabulated in Tables 5 and 6 and analyzed in the following section.

14 $$\begin{aligned} MSE= {\frac{1}{mn}}{{\sum \limits _{i=0}^{m-1}}{\sum \limits _{j=0}^{n-1}}}{\left[ Y(i,j)-X(i,j)\right] }^{2} \end{aligned}$$

where X and Y represent enhanced and input images, respectively; m and n represent the number of rows and columns.

15 $$\begin{aligned} PSNR= 10 log_{10} \left( \frac{R^2}{MSE} \right) \end{aligned}$$

where R represents the maximum fluctuation present in the input image.

16 $$\begin{aligned} SSIM(x,y)= l(x,y) * c(x,y) * s(x,y) \end{aligned}$$

where l(x,y), c(x,y), s(x,y) represents luminance, contrast and structure comparison between input(x) and enhanced(y) images.

Pearson’s correlation coefficient, $r_{1}$

17 $$\begin{aligned} r_1= \frac{{\sum }_{i}\left( x_i - x_m\right) \left( y_i - y_m\right) }{{\sqrt{{{\sum }_{i}\left( x_i - x_m\right) ^2}}}{\sqrt{{{\sum }_{i}\left( y_i - y_m\right) ^2}}}} \end{aligned}$$

where $x_i$ and $y_i$ Denote $ith$ pixel intensity of images 1 and 2, respectively $x_m$ and $y_m$ correspond to the mean intensity of images 1 and 2.

Shannon entropy, H(X)

18 $$\begin{aligned} H(X)= - {\sum }_{i}p(x_{i})*log_{2}p(x_{i}) \end{aligned}$$

where p($x_i$ ) represents normalized histogram frequency of pixels in input image- X.

Universal Quality Index, UQI

19 $$\begin{aligned} UQI= {\frac{{\sigma }_{xy}}{{\sigma _{x}}{\sigma _{y}}}} *{\frac{2x_{m}y_{m}}{\left( x_{m}^2 + y_{m}^2\right) }} * {\frac{2{\sigma }_{x}{\sigma }_{y}}{\left( {\sigma }_{x}^2 + {\sigma }_{y}^2\right) }} \end{aligned}$$

**Table 5:** MSE, PSNR, and UQI of RFMiD training dataset.

Category	MSE	PSNR	UQI	Category	MSE	PSNR	UQI
DR	1920.08	29.79	0.79	ODP	1299.34	29.43	0.82
ARMD	1435.56	29.10	0.83	ODE	1744.64	28.45	0.81
MH	2307.89	28.81	0.77	ST	1378.98	29.68	0.83
DN	1847.04	28.99	0.80	AION	1892.61	28.85	0.80
MYA	1520.44	29.34	0.81	PT	1921.71	30.00	0.78
BRVO	1706.45	29.07	0.82	RT	1430.28	29.50	0.83
TSLN	1785.90	29.01	0.80	RS	1051.56	28.57	0.85
ERM	1142.40	28.92	0.83	CRS	1335.30	28.74	0.83
LS	1629.01	29.65	0.81	EDN	1522.62	29.29	0.78
MS	1073.13	28.63	0.85	RPEC	1260.10	29.33	0.83
CSR	1536.91	28.66	0.82	MHL	1809.01	29.21	0.81
ODC	1462.94	29.28	0.82	RP	864.15	28.77	0.86
CRVO	2290.57	28.92	0.79	OTHER	1579.63	29.04	0.83
TV	1541.22	28.96	0.83	HF	2090.18	29.18	0.78
AH	1707.45	29.29	0.82	Total Average	1589.21	29.12	0.81

**Table 6:** Shannon entropy of Input image, Enhanced Image, SSIM, Pearson Correlation of Coefficient of RFMiD training dataset.

Category	H(x) of Input	H(x) of Output	SSIM	r1	Category	H(x) of Input	H(x) of Output	SSIM	r1
DR	5.57	5.87	0.76	0.91	ODP	5.77	6.09	0.79	0.94
ARMD	5.91	6.10	0.78	0.94	ODE	5.98	6.27	0.77	0.95
MH	5.72	5.92	0.77	0.95	ST	5.85	5.88	0.80	0.93
DN	5.70	6.01	0.77	0.95	AION	5.81	6.04	0.77	0.95
MYA	5.95	5.95	0.77	0.93	PT	5.60	5.93	0.76	0.94
BRVO	5.77	6.11	0.78	0.95	RT	5.79	6.10	0.80	0.96
TSLN	5.82	5.94	0.76	0.92	RS	6.05	6.42	0.80	0.96
ERM	5.88	6.30	0.80	0.96	CRS	5.98	6.36	0.79	0.95
LS	5.68	6.07	0.78	0.94	EDN	5.68	6.00	0.75	0.88
MS	5.96	6.32	0.81	0.96	RPEC	5.79	6.22	0.79	0.96
CSR	5.81	6.20	0.77	0.96	MHL	5.86	6.15	0.78	0.95
ODC	5.76	6.07	0.78	0.95	RP	6.17	6.54	0.82	0.96
CRVO	5.81	6.12	0.76	0.95	OTHER	5.89	6.21	0.79	0.95
TV	5.69	6.06	0.78	0.95	HF	5.75	6.11	0.73	0.93
AH	5.93	6.18	0.78	0.95	Total Average	5.82	6.12	0.78	0.94

**Figure 4:**
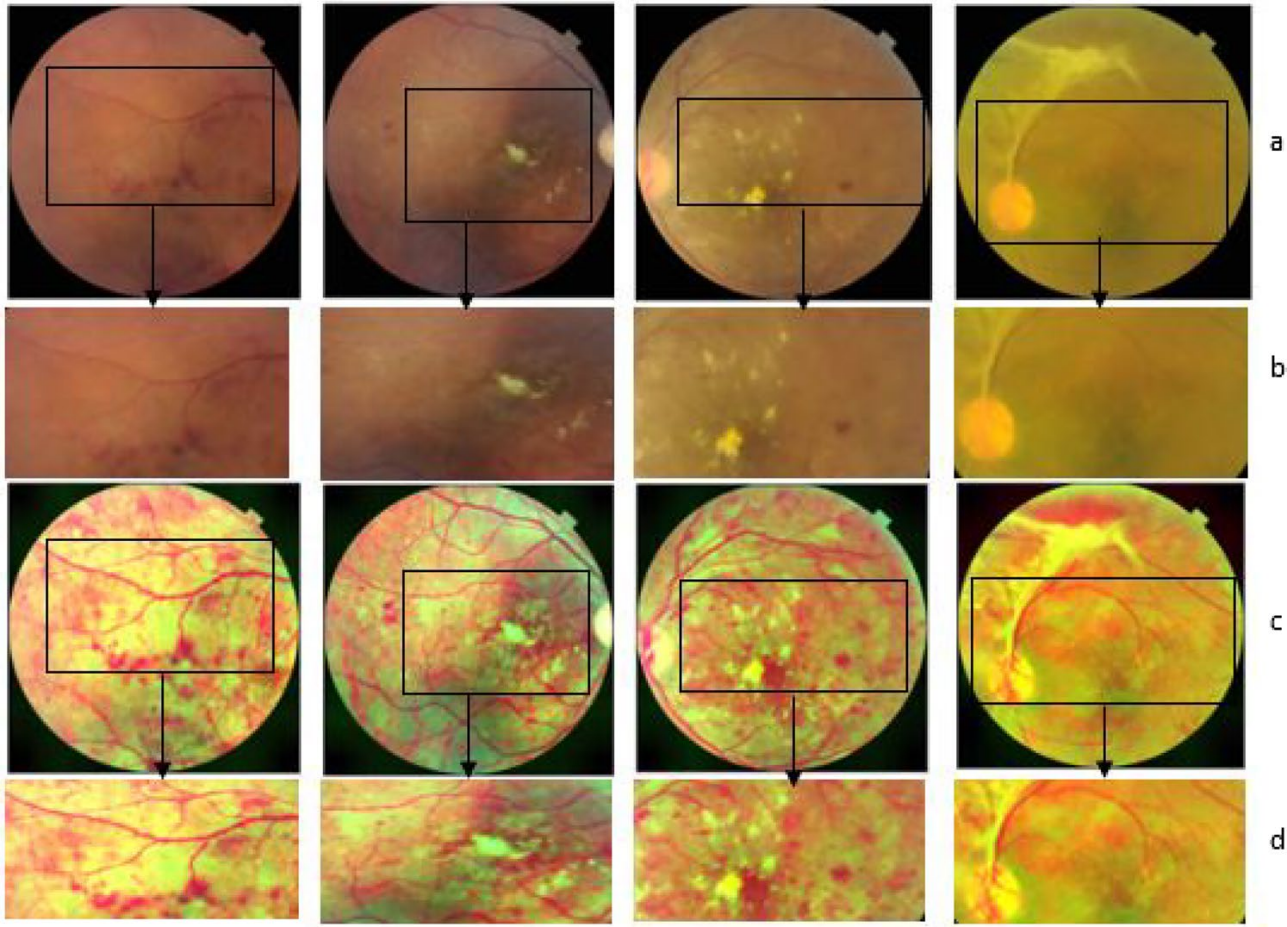
(**a**) Input image, (**b**) A section of the input image, (**c**) Image enhanced using the proposed technique, (**d**) A section of enhanced image.

**Table 7:** Average PSNR and SSIM measure comparison of MESSIDOR dataset.

Methods	PSNR	SSIM
Zhou et al.^7^	23.11	0.58
Gupta et al.^15^ (for q=3)	27.67	0.66
Gupta et al.^15^ (for q=5)	28.40	0.69
**Proposed**	29.77	0.63

**Table 8:** Average PSNR and Entropy measure comparison of DRIVE dataset.

Methods	PSNR	Entropy
Mohammed et al.^16^	24.42	5.63
Navdeep et al.^17^ (RIHE-RVE)	29.33	5.71
**Proposed**	29.67	5.61

**Table 9:** Average PSNR and Entropy measure comparison of MESSIDOR and DRIVE datasets.

Methods	PSNR	Entropy
Qureshi et al.^18^	23.78	4.60
**Proposed**	27.79	7.07

**Table 10:** Results of VGG16 model.

Group	Validation accuracy (%)	Test accuracy (%)	F1 score
Input images with the proposed enhancement technique	**84.06**	**89.53**	**0.89**
Input images without performing enhancement	82.34	87.81	0.86

## Discussion

We evaluated the performance of proposed enhancement technique using the performance measures- MSE, PSNR and UQI. Table 5, displays MSE, PSNR, and UQI values of each retinal disease categories. Out of all the disease categories, fundus images with Retinitis Pigmentosa (RP) achieved the lowest MSE value of 864.15, a PSNR value of 28.77, and a higher UQI value of 0.86. UQI above 0.85 is achieved in images with the following abnormalities - Macular Scar (MS), Retinitis (RS), and Retinitis Pigmentosa (RP). Parafoveal Telangiectasia (PT) achieved a higher PSNR value of 30.00. All three abnormalities are related to anomalies or inflammations in arteries, retinal haemorrhage, etc. The MSE values of the above disorders are less in the range of 1000s compared to CRVO, having an MSE value of 2290.57, and the PSNR value is higher than 28.

UQI of 0.83 with SSIM above 78% and Pearson correlation of Coefficient above 93% is achieved on enhancing the images with retinal disorders related to the choroid, features in the fovea, vessels - ARMD, ST, RT, ERM, CRS, RPEC, TV. The MSE value of these retinal disorders ranges from 1142.40 to 1579.63, and the PSNR value is between 28.74 and 29.68. For diseases with symptoms in optic disc (ODP, ODE, ODC), a UQI of 0.81 or 0.82 is achieved with PSNR value in the range 28.45 to 29.43 and Pearson correlation of Coefficient minimum 94%. The MSE value of Media Haze is higher than other retinal abnormalities. The UQI metric is between 0.77 and 0.79 for the retinal diseases- DR, MH, CRVO, PT, and EDN with MSE values between 1920.08 and 2307.89 except for EDN with MSE values equal to 1522.62. For other retinal anomalies like - DN, MYA, BRVO, TSLN, LS, CSR, MHL, and AH the UQI metric is between 0.80 and 0.82; the PSNR values are above 29.0 except for CSR and DN with PSNR 28.66 and 28.99. From the analysis, it can be inferred that high UQI is achieved for retinal images with artery or vessel inflammation-related disorders like RP, RS, and MS by maintaining SSIM of 80% and above with a Pearson correlation of Coefficient 96%. The overall Pearson correlation of Coefficient for all the disease categories is above 90% except for Exudation with 88%. A minimum of 73% structural similarity is maintained between the input image and enhanced images. The tabulated results show that the proposed enhancement method achieves an overall average UQI of 0.81 and PSNR of 29.12 by enhancing the features of the input image from an average Shannon entropy of 5.82 to 6.12 by maintaining average 78% structural similarity and 94 percent Pearson correlation coefficient between the input and enhanced image.Figure 3. compares samples of the input image and the enhanced image in color scale and grayscale. The features are well-enhanced and prominent in the enhanced image compared to the original image. From the closer sectional view shown in Figure 4, it is evident that anomalies present in the path of retinal vessels, like red haemorrhages, are enhanced well. The path of blood vessels serves as vital evidence to identify diseases like retinal pigment epithelium. Figure 4 shows proliferated retinal vessels in the optic cup better in the enhanced retinal image compared to an original input image. It is challenging to enhance both retinal vessels and the optic cup in a fundus image because either of them gets suppressed while enhancing the other. The advantage of the proposed method is it is efficient in enhancing the vessel as well as the optic cup for an image of different resolutions.

We tested the suitability of the proposed enhancement technique on two other publicly available datasets - MESSIDOR and DRIVE. Table 7, 8 and 9 presents the performance comparison of the proposed technique with other existing enhancement methods in terms of PSNR, SSIM, and Entropy values. The results show that the proposed enhancement technique based on color dominance outperforms other considered techniques by achieving a higher PSNR value. Further, we evaluated how best our proposed technique helps to identify the presence or absence of disease in the RFMiD dataset. From Table 10, it can be inferred that the presence or absence of retinal abnormality detection with the VGG16 model using the proposed enhancement technique can achieve 89.53% test accuracy, 0.89 F1 scores compared to the input images without enhancement achieving 87.81% test accuracy, and 0.86 F1 score.

## Conclusion

Retinal image enhancement is an essential step under the pre-processing stage to better view the retinal anomalies for identifying the type of disease a patient suffers. This paper proposes an efficient retinal image enhancement technique based on color dominance in an image. In stage 1, the variance of the blue channel is calculated to find the color dominance of the input image. Depending on the resultant value, a* or b* channel of Lab color space is chosen for enhancement. If the variance value is less than the threshold, the information in the blue channel is less. So, a* channel of Lab color space is chosen for enhancement. For values above the threshold, b* channel of Lab color space is chosen for enhancement. The Contrast limited adaptive histogram equalization (CLAHE) technique is applied to perform an enhancement on the selected channels. Enhanced image in Lab space is passed to stage 2. In stage 2, the corresponding green channel is enhanced, followed by noise removal using a bilateral filter and auto-optimizing brightness and contrast.

The proposed image enhancement technique is analyzed using the metrics - UQI, MSE, PSNR, Shannon entropy, SSIM, and Pearson Correlation of Coefficient on the training set of the RFMiD dataset. The analysis shows that enhancement of images with retinal diseases - Macular Scar, Retinitis, and Retinitis Pigmentosa achieved the highest UQI of 0.85 and above. The overall average UQI obtained by evaluating 1920 train images is 0.81 with a 27.90 PSNR value, 1757.6 MSE value with 75% structural similarity, and 96% Pearson correlation of Coefficient. Further, the performance of the proposed enhancement technique in identifying the presence or absence of retinal disease is analyzed using RFMiD data-set and the pre-trained VGG16 deep learning model. The model trained with training images enhanced with the proposed enhancement technique achieved 89.53% test accuracy and an F1 score of 0.89 on RFMiD test set images. The model trained using images without performing any enhancement achieved 87.81% test accuracy and an F1 score of 0.86. The proposed enhancement technique is evaluated on MESSIDOR and DRIVE datasets as well. The results show that the proposed enhancement techniques outperform other considered methods by achieving higher PSNR values.

## Data Availability

The dataset used in this research is Retinal Fundus Multi-Disease Dataset. It is available in the online repository - https://ieee-dataport.org/open-access/retinal-fundus-multi-disease-image-dataset-rfmid.
